# Alpha-Synuclein and Mitochondrial Dysfunction in Parkinson’s Disease: The Emerging Role of VDAC

**DOI:** 10.3390/biom11050718

**Published:** 2021-05-11

**Authors:** Pierpaolo Risiglione, Federica Zinghirino, Maria Carmela Di Rosa, Andrea Magrì, Angela Messina

**Affiliations:** 1 Department of Biomedical and Biotechnological Sciences, University of Catania, Via Santa Sofia 64, 95125 Catania, Italy; pierpaolo.risiglione@phd.unict.it (P.R.); federica.zinghirino@phd.unict.it (F.Z.); mcdirosa@unict.it (M.C.D.R.); 2 Department of Biological, Geological and Environmental Sciences, University of Catania, Via Santa Sofia 64, 95125 Catania, Italy; andrea.magri@unict.it; 3 we.MitoBiotech S.R.L., C.so Italia 172, 95125 Catania, Italy

**Keywords:** Alpha-Synuclein, mitochondria, VDAC, Parkinson’s disease, mitochondrial dysfunction

## Abstract

Alpha-Synuclein (αSyn) is a protein whose function is still debated, as well as its role in modulation of mitochondrial function in both physiological and pathological conditions. Mitochondrial porins or Voltage-Dependent Anion Channel (VDAC) proteins are the main gates for ADP/ATP and various substrates towards the organelle. Furthermore, they act as a mitochondrial hub for many cytosolic proteins, including αSyn. This review analyzes the main aspects of αSyn-mitochondria interaction, focusing on the role of VDAC and its emerging involvement in the pathological processes.

## 1. Introduction

Parkinson’s disease (PD) is the second most common age-related neurological disorder that affects 2–3% of the population over 65 years of age [1]. Clinical diagnosis relies on the presence of motor symptoms including bradykinesia, tremor at rest, and rigidity [2]. PD is a multifactorial disease arising from different factors including environmental, aging, genetics or a combination of all or several of them. The pathological event leading to the motor symptoms manifestation is mainly represented by the death of dopaminergic (DA) neurons in the substantia nigra pars compacta (SNpc). This event causes the subsequent dopamine depletion in the striatum and the impairment of the thalamo-corticobasal ganglia circuits [3,4,5]. Beyond the motor symptoms, patients with PD develop several non-motors features, including disorders of sleep-wake cycle regulation, autonomic dysfunction, hyposmia, depression, fatigue, pain, and cognitive impairment. Some of these effects characterize a prodromal phase that precedes the disease onset, while others appear during the development of motor symptoms in the early, mild or late PD stage [6].

The physiological dopamine precursor, L-DOPA, is still the gold standard for PD treatment, being used as a valid substitute for striatal dopamine loss [7]. Despite its remarkable effectiveness in treating motor symptoms treatment, L-DOPA therapy is associated with long-term side effects including the development of motor complications, known as L-DOPA induced dyskinesia [8]. Moreover, L-DOPA does not counteract the non-motor symptoms which are sometimes as disabling as the motor ones [9]. To date, there are no treatments that can prevent, block or slow PD symptoms progression.

Along with the loss of DA neurons, the appearance of Lewy bodies (LBs) represents the main pathological hallmark of the disease [10]. LBs are cytoplasmatic neuronal inclusions in which the small protein alpha-synuclein (αSyn) represents the main component [11]. During a complex misfolding process, which is still not fully understood, αSyn aggregates into amyloid fibrils that accumulate intra-cellularly [12]. Notably, αSyn pathology is a feature shared by several disorders generally known as *synucleinopathies*.

Mitochondrial dysfunction has also been strongly implicated in the pathogenesis of PD. As mitochondria are indispensable for sustaining the high energy demand of neurons, an in-depth understanding of PD-associated mitochondrial pathogenetic mechanisms is crucial for developing new therapeutic strategies. In addition to their role in ATP production, mitochondria participate in the regulation of programmed cell death, calcium homeostasis and many other essential cellular processes [13]. In this perspective, the emerging role of mitochondrial porins, better known as Voltage-Dependent Anion-selective Channels (VDAC) deserves particular attention.

VDACs are pore-forming proteins located in the outer mitochondrial membrane (OMM) where they account for ~50% of the overall protein content [14,15,16] and are responsible for ~90% of the OMM’s overall permeability [17]. Playing such an important role, VDAC is considered the ‘governor’ of mitochondrial function [18]. Its main function is to maintain the metabolic cross-talk between the mitochondria and the rest of the cells, allowing the passive diffusion of essential hydrophilic metabolites up to 5 kDa in size [19,20]. Furthermore, recent literature depicts a clear involvement of VDAC in the onset of various neurodegenerative diseases, including PD [21].

The aim of this review is to collect the available information about the αSyn–VDAC relationship, as well involvement in PD-associated mitochondrial dysfunction. Emphasis has also been placed on possible therapeutic approaches aimed at counteracting the toxic effects of αSyn at the mitochondrial level.

## 2. Structure of αSyn and Its Amyloid Properties

αSyn is a small protein of 14 kDa that is ubiquitously expressed, mainly present in the presynaptic terminals of the central nervous system, where it regulates neurotransmitter release and synaptic plasticity [22]. Although αSyn is also found in other subcellular compartments, such as nucleus [23], mitochondria [24], and endoplasmic reticulum (ER) [25], the significance of this different localization has not been fully elucidated. In 1997, several point mutations in the gene encoding αSyn, SNCA, were associated with early-onset forms of PD [26]. In the same year, αSyn was also identified as the main component of LBs [11], highlighting its central role in PD pathogenesis.

αSyn is a 140 amino acids polypeptide with three distinct functional domains. The N-terminal region forms an amphipathic α-helix structure that is responsible for the interaction of αSyn with lipid membranes [27]. The central domain, known as the non-amyloid-β component (NAC), was first identified in the senile plaques of Alzheimer’s disease brains [28] and contains a highly hydrophobic motif essential for αSyn aggregation [29]. The C-terminal domain, rich in negatively charged amino acids and proline residues, is a region rich in post-translational modifications (PTMs), including phosphorylation at the Ser 129 (S129), first detected in LBs extracts from PD patients [30]. Interestingly, phosphorylation of S129 modulates the binding of αSyn to lipid membranes and enhances its interaction with metal ions and other proteins, thus promoting protein aggregation [31,32].

The native αSyn is an intrinsically disordered protein lacking a well-defined rigid structure and with a still controversial physiological conformation. It has been proposed that the protein exists as an unfolded monomer in a dynamic equilibrium between a cytosolic and a membrane-bound α-helical state [33]. Nevertheless, αSyn has been isolated in several conformational states, mainly as a tetramer, from various cell types [34,35]. During its pathological misfolding process, monomeric αSyn acquires a β-sheet-rich structure and self-assembles into metastable intermediate oligomers before becoming fibrils that accumulate within LBs. The aggregation process leads to the formation of a wide variety of αSyn amyloid fibrils characterized by a typical cross-β organization [36]. Differences in structural, biochemical and pathological features of the fibrils are probably linked to the heterogeneous spectrum of clinical signs observed in synucleinopathies (e.g., PD, dementia with LBs, and multiple system atrophy) [37,38,39].

The propensity of αSyn to form fibrils and inclusions can be influenced by several factors. These include missense mutations (best known: A53T, E46K, A30P) [40], gene duplication and triplication that increase protein expression level [41], but also environmental toxins [42], oxidative stress [43], PTMs [44], and high metal concentration [45]. Fibrillar α-Syn species have been proposed to contribute to neurodegeneration by perturbing cellular ion homeostasis [36], cellular proteostasis [46,47] and by compromising the function of various organelles [48]. Furthermore, depending on the disease stage, α-Syn aggregates have been found in different brain regions of PD patients suggesting a prion-like cell-to-cell transmission [49,50,51]. To this end, the exogenous addition of preformed αSyn fibrils (PFFs) to cells in culture recapitulates the self-assembly process of endogenous αSyn into amyloid fibrils [52,53]. Similarly, injections of PFFs into WT mice and non-human primates induce progressive synucleinopathy [54,55]. Taken together, these and other data suggest that when exogenously added, αSyn fibrils are taken up by neurons and act as “seeds” (i.e., conformational template) for the aggregation of endogenous monomeric αSyn [53]. However, some issues have to be taken into account. For instance, the data on αSyn propagation have been obtained in animal models but not humans. Another apparent discrepancy is linked at the different rate of progression between PD and prions [56].

Recently, the α-Syn toxicity has also been strongly associated with the formation of oligomeric species. Data from studies of post-mortem PD tissues suggest that the neurotoxicity of soluble αSyn oligomers arises from their ability to both modify the stability of biological membranes and interfere with the function of mitochondrial proteins [57]. Thus, αSyn oligomers cause a cascade of toxic effects such as membrane permeabilization, increased Ca^2+^ influx, mitochondrial dysfunction, loss of proteostasis, oxidative stress, and cell death [58,59,60,61]. Overall, the relative contribution of oligomeric and fibrillar αSyn species to neurotoxicity in PD remains a subject of intense and controversial debate in the field and deserves further investigations.

## 3. Oxidative Stress: A Key to Understanding the DA Neuron Vulnerability in PD

Aging represents one of the main risk factors for PD and other neurodegenerative disorders [62]. It is known that age-related biochemical and molecular changes occur in nigral DA neurons leading to mild mitochondrial dysfunction, iron and calcium dysregulation and antioxidant deficiencies [63,64]. However, when aging occurs along with additional risk factors associated with PD, the probability of developing the disease increases. By their nature, DA neurons are subjected to high basal levels of oxidative stress, which leads to reactive oxygen species (ROS) and pro-oxidant dopamine o-quinones production [65,66,67]. A potentially dangerous impairment in neuronal redox balance is observed in PD patients. In this case, oxidative stress is the result of an impairment in physiological ROS production and in the ability to counteract them. Furthermore, oxidative stress is enhanced by iron accumulation in SNpc. Iron has a crucial role in this context since it takes part in the Fenton reaction in which H_2_O_2_ produced by enzymatic or oxidative dopamine metabolism reacts with labile ferrous iron (Fe^2+^) to generate damaging hydroxyl radicals [67,68]. Notably, in SNpc of PD patients, iron levels are twofold higher than age-matched controls, although the precise process behind this increase is still unknown [69,70].

The vulnerability of SNpc to oxidative insults can also be explained through an age-dependent reduction in the levels and function of antioxidant enzymes, such as superoxide dismutase, glutathione peroxidase and glutathione reductase [64,71,72]. This is what emerges from the comparison between post-mortem SNpc of healthy aged and younger individuals. This deficiency suggests a gradual decrease in the capability of DA neurons to face rising ROS production. Furthermore, oxidative stress signals alongside other pathological stressors (i.e., protein aggregation, gene mutations, environmental factors) can result in a neuro-inflammatory process mediated by glial activation. Under these pathological conditions, activated microglia and reactive astrocytes release inflammatory mediators, which contribute to the degeneration of DA neurons [73].

Data here reported depict a crucial involvement of oxidative stress and neuroinflammation for DA neurons degeneration in PD.

## 4. Mitochondrial Dysfunction in Parkinson Disease: A Brief Overview

Mitochondria are essential organelles for cell life. They represent the main producer of cellular energy in the form of ATP and control many other important physiological processes [74]. Mitochondria are also the main cellular source of ROS. During mitochondrial activity, superoxide radical is generated as the main by-product of the ATP synthesis due to premature electron transfer from complexes I and III of the electron transport system (ETS) to O_2_ [75,76]. For this reason, dysregulated mitochondrial homeostasis is one of the causative factors of aging and neurodegenerative diseases, such as amyotrophic lateral sclerosis (ALS), Alzheimer’s and Parkinson’s diseases. Both environmental and genetic factors contribute to the mitochondrial dysfunction typically associated with PD. For example, chronic exposure to environmental toxins represents a well-established risk factor for the disease onset and many of these compounds exert their toxicity primarily at the mitochondrial level.

MPTP (1-methyl-4-phenyl 1,2,3,6-tetrahydropyridine) is a neurotoxic contaminant of an illicit opioid preparation able to cross the blood-brain barrier [77]. After oxidation to its active form MPP^+^, it selectively damages DA neurons by blocking complex I (NADH ubiquinone oxidoreductase) of ETS [78]. The main consequence is a dramatic depletion of ATP produced by the organelle and an increase in ROS [78,79]. However, the damage induced by this neurotoxin seems to be much more extensive. For instance, MPP^+^ decreases mitochondrial gene expression and alters the level of mitochondrial proteins [80,81]. Recent work by our group shows that exposure of differentiated SH-SY5Y cells to a sublethal dose of MPP^+^ severely compromises the overall respiratory profile of these cells, especially affecting the stability and function of the inner mitochondrial membrane (IMM) [82]. Like MPTP, rotenone, and Paraquat, commonly used as pesticides and herbicides respectively, induce the loss of DA neurons by decreasing the activity of complex I and causing oxidative stress in a similar way to MPP^+^ [83,84]. Furthermore, a 30% reduction in complex I activity has been found in the brains of PD patients, reinforcing the link between complex I inhibition and pathology [85,86].

In 5–10% of cases, PD occurs as heritable disease caused by pathogenic mutations in specific genes associated with dominant or recessive forms of familial PD [87]. The first mutations identified as causing familial PD were in the SNCA gene encoding for αSyn [26], whose relationship to the mitochondrion will be further discussed. Other PD-associated genes are found in loci referred to as PARK. Not surprisingly, most of these encode proteins strictly related to mitochondrial physiology. PTEN-induced putative kinase 1 (PINK1) and Parkin are key regulators of the mitochondrial quality control system and their functions are largely linked to each other, especially in the induction of the mitophagy process [88]. Briefly, PINK1 is a serine/threonine kinase that collaborates with the E3 ubiquitin ligase Parkin to tag damaged mitochondria and target them for degradation [89,90]. Several mutations in both Parkin and PINK1 compromise mitophagy and support the involvement of this degradation pathway in PD pathogenesis, although further studies are needed to elucidate whether impairment of this pathway contributes to neuronal death [91,92,93].

Altogether, data collected here strengthen the relationship between mitochondrial dysfunction and PD pathogenesis.

## 5. αSyn and Mitochondria: Between Harm and Well-Being

αSyn is a predominantly cytosolic protein whose function is only partially known. The physicochemical features of the N-terminal domain of αSyn give the protein a high affinity for mitochondrial membranes [94]. In addition, the same domain contains a cryptic mitochondrial targeting sequence with a 32 amino acid stretch that appears to be critical for mitochondrial translocation [95]. In mitochondria, αSyn has been found in all compartments where, in its native and unfolded state, it appears to regulate some organelle functions. Recently, a physiological involvement of αSyn in mitochondrial dynamic has been suggested [96]. These processes are controlled by the mitochondrial quality control system (mitophagy) which, following mitochondrial fission and fusion, allows the cell to maintain the correct number of functional mitochondria. In a fission process, a single organelle splits into two or more, whereas fusion leads to a reduction in the number of mitochondria. These actions aim to regulate the shape, size and number of mitochondria in response to both changes in the cellular environment and mitochondrial damage [97]. It has been reported that αSyn favors mitochondrial fission events while inhibiting fusion through the activity of Mfn1, Mfn2, and Opa1. This may promote the formation of small mitochondria to facilitate organelle mobilization across the axons of DA neurons [96].

Controlling mitochondrial bioenergetic is another crucial function performed by αSyn. In mice lacking αSyn, a reduction in the activity of ETS complexes I and/or III was observed and it was associated with abnormalities in mitochondrial lipid composition [98]. Moreover, Ludtmann and colleagues reported that mitochondria-derived from α, β and γ synuclein knock-out mice were characterized by uncoupled mitochondrial respiration in which electron flow through the ETS enzyme was only partially associated with the ADP phosphorylation process [99]. In the same work, the addition of monomeric αSyn to isolated brain mitochondria increased ATP production through a direct interaction between αSyn and the α-subunit of ATP synthase. These findings strengthen the important role of monomeric αSyn as mitochondrial bioenergetic regulator [99].

In contrast to monomers, αSyn oligomers are believed to be deleterious for ATP synthesis through a mechanism that increases ROS production, leading to oxidation of lipids and the β subunit of ATP synthase [100]. The transition of αSyn from monomeric to pathological aggregated species is known to be accompanied by mitochondrial dysfunction, especially in PD [101]. It is also interesting to note that αSyn has been detected in the mitochondria of the SNpc and striatum of both non-PD and PD subjects, although pathological accumulation of the protein within the organelle has been found exclusively in PD patients [95].

The mechanism by which αSyn reaches the intermembrane space (IMS) and other compartments is still a matter of debate, although several hypotheses have been proposed. The β-barrel proteins of the OMM are a group of conserved integral membrane proteins characterized by an amphipathic structure in which polar residues are oriented towards hydrophilic compartments while non-polar ones are facing the hydrophobic environment of the lipidic membrane [102]. Several of them have been indicated as the import route of αSyn. For instance, TOM40 is the main component of the translocation of outer membrane (TOM) complex, the evolutionarily conserved import pathway exclusively dedicated to mitochondrial protein precursors synthetized in the cytosol. In vitro and in vivo studies show that anti-TOM40 antibodies abolish the αSyn translocation across the OMM [95]. VDAC has also been proposed as a possible route for αSyn uptake into the mitochondria. It has been demonstrated that αSyn can translocate through VDAC1 pore at high voltage in electrophysiological experiments [103]. Furthermore, Rovini and colleagues found a significant reduction of αSyn localization at the IMM in cells with low levels of VDAC1 [104]. However, further studies are needed to understand which specific cellular conditions promote the αSyn translocation inside the mitochondrion.

Regardless of its gateway, under pathological conditions, the accumulation of aggregated αSyn in mitochondria reduces complex I activity which, in turn, leads to increased ROS production and mitophagy [58,105,106,107]. A growing body of evidences supports the ability of αSyn oligomers to affect many essential mitochondrial processes. Notably, through interaction with TOM20, another regulatory subunit of the TOM complex, oligomeric αSyn impairs the import of mitochondrial proteins encoded by the nuclear genome [108]. This has also been observed in other neurological disorders. In ALS, mutated forms of the antioxidant enzyme Cu/Zn superoxide dismutase (SOD1) aggregated on the cytosolic surface of mitochondria where they affect the overall protein content of the organelle [109]. The association of oligomeric αSyn with mitochondria has been linked to decreased mitochondrial sirtuin 3 levels, as well as organelle biogenesis, leading to an overall alteration in mitochondrial dynamics [110].

The emerging framework clearly indicates a different effect of monomeric and aggregated αSyn on mitochondrial functionality.

## 6. VDAC: A Key Crossroads for the Mitochondrial Functionality

**Figure 1:**
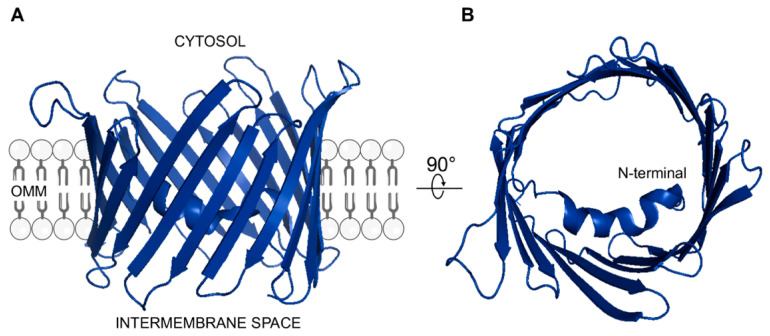
3D structure of human VDAC1 (**A**) Lateral view showing the β-barrel organization. The protein is embedded in the OMM facing hydrophilic residues towards cytosolic or mitochondrial IMS compartment. (**B**) Top view showing the α-helix structured N-terminal domain located within the channel lumen. Both structures were drawn by using PyMOL software (Molecular Graphics System, version 2.4.1, 2021) and the available PDB structure (ID: 2JK4) as template.

VDAC proteins are a family of membrane channels embedded in the OMM that completely cover the membrane giving it the typical sieve-like aspect [111]. X-ray crystallography and NMR studies contributed to determining the 3D structure of the VDAC channel. As shown in Figure 1, the pore is organized in a β-barrel consisting of 19 anti-parallel β-strands, except for the N-terminal domain which is arranged in a α-helix structure located inside the barrel [112,113,114]. It has been proposed that the N-terminus may be mobile and partially exposed toward the cytosolic compartment where it mediates protein–protein interactions [115,116].

Given their pore structure and abundance, VDAC proteins allow metabolite transport across the OMM. In particular, VDAC represents the main route for newly synthesized mitochondrial ATP, and the entry route to the organelle for cytosolic ADP, pyruvate, glutamate, succinate, and Krebs’s cycle intermediates [20,117]. Ions, including Na^+^, Cl^–^, K^+^ and Ca^2+^, as well as NAD^+^/NADH also use VDAC as mitochondrial gateway [13].

In humans, and more generally in mammals, three different isoforms of VDAC are expressed, named VDAC1, VDAC2 and VDAC3 [118]. VDAC1 is the most highly expressed isoform and overcomes VDAC2 and VDAC3 by one and two orders of magnitude respectively [119]. Despite high similarity in terms of gene organization, protein sequence and structures, VDACs differ in both tissue-specific expression and for the presence of isoform-specific function(s). This is also reinforced by the distinct transcriptional regulators and protein binding partner associated with each of them [120,121]. The transcription factor binding sites (TFBSs) identified on the VDAC1 promoter confirm its general role in mitochondrial functionality. The VDAC2 promoter contains TFBSs involved in organogenesis and neurodevelopment, whereas the VDAC3 one is especially rich in binding sites for regulators of germinal development and sex determination [120,122]. In addition, VDAC1 is mainly responsible for regulating metabolic flows through the OMM, whereas VDAC3 has been proposed as a mitochondrial sensor of oxidative stress [123]. This is what emerges from the recently described specific set of PTMs [124,125,126] and the analysis of the VDAC3 interactome [127].

Since their identification, the activity of VDACs has been studied after incorporation into artificial membrane (known as planar lipid bilayers, PLB) by using electrophysiological techniques. The main isoform, VDAC1, shows a high propensity to form pores into PLB [128,129,130]. These pores are characterized by peculiar conductance values that vary in accordance with the voltage applied. At low potential, close to 0 mV and between ±10 mV, VDAC1 is in a high-conductance state, also known “open state”. However, when the voltage increases in both positive and negative directions, starting from ±10–30 mV, channel conductance switches towards several low-conductance or “closed states” [128,129,130]. Ion selectivity is similarly affected by the applied voltage. Indeed, while in the open state VDAC1 is slightly selective for anions over cations, in the closed state it becomes slightly cation-selective [128,129,130]. Similar electrophysiological features have been observed for all the other human VDAC isoforms (even if some peculiarity in the case of VDAC3 [128]) and for porins from yeast, fruit-fly, plants and other mammals [131,132,133,134,135].

Due to their strategic position at the interface between cytosol and mitochondria, VDACs serve as a binding site for many crucial proteins. The best-known partner are the metabolic enzymes families of hexokinases (HKs), glycerol kinases, glucokinases and creatin kinases [136,137]. In particular, HKs catalyse the first and rate-limiting reaction of glycolysis, and through interaction with VDAC1, they take advantage of direct access to newly synthetized ATP. Not surprisingly, in many tumors both VDAC1 and HKs are overexpressed [138]. The increased level of VDAC1/HKs complexes correlates with an increased glycolytic rate by which cancer cells tend to promote glycolytic metabolism over mitochondrial respiration even in the presence of oxygen. This phenomenon, known as the Warburg effect, is intended to promote tumor cell proliferation [139,140]. Members of the Bcl-2 family proteins, key regulators of apoptosis, have also been listed as crucial VDAC binding partners. These interactions are important in regulating the OMM permeability, as will be discussed below.

In addition to the physiological interactors pattern, VDACs, and in particular VDAC1, act as a docking site for misfolded or mutated proteins associated with many neurodegenerative disorders, including PD. Interaction with these abnormal proteins alters the physiological activity of VDAC, contributing to the mitochondrial dysfunction typical of these pathologies (as reviewed in [21,141]). In Alzheimer’s disease, β-amyloid peptide and hyperphosphorylated tau co-precipitate with VDAC1 in brains patients [142]. Moreover, the addition of β-amyloid monomers or oligomers to VDAC1 reconstituted in PLB decreased or enhanced the conductance of VDAC1, respectively [143]. A similar co-precipitation was observed in ALS affected tissues, involving VDAC1 and mutated SOD1 forms (i.e., G93A, G85R) [144]. In this specific case, this interaction VDAC1/SOD1 mutant reduced ADP flux across VDAC in mitochondria isolated from the spinal cord mitochondria of transgenic SOD1 rats [144]. Furthermore, several SOD1 mutants were found to specifically modulate VDAC1 conductance at PLB in both open and closed state [144,145,146].

## 7. VDACs Are Crucial Players in Apoptosis Regulation

The mitochondrial homeostasis is strictly controlled and malfunctioning mitochondria are cleared by mitophagy, a specific form of autophagy that is impaired in PD hereditary forms. However, when mitochondrial stress produces irreparable cellular damage, apoptosis is initiated through the activation of pro-apoptotic proteins of the Bcl-2 family located or recruited to OMM. Indeed, mitochondria contain the so-called apoptogenic factors (cytochrome c, AIF, Smac/Diablo) which usually localize into the IMS. Nevertheless, several stimuli can perturb the permeability of the OMM leading to their release into the cytosol and the activation of the caspase cascade [147].

Bax and Bak are two pro-apoptotic members of the Bcl-2 family proteins, indispensable for OMM permeabilization during mitochondrial apoptosis. Under physiological conditions, Bax is mainly located in the cytosol, in contrast to Bak which constantly resides in OMM in the inactive conformation. In the case of increased levels of oxidative stress and cytosolic Ca^2+^, as well as DNA damages, Bax undergoes a conformational change that increases its affinity for OMM [148,149]. As a consequence of this, Bax translocates from the cytosol to the mitochondria where it oligomerizes or promotes the assembly of hetero-oligomers with Bak and/or VDAC1 [150,151]. These events lead to the OMM permeabilization and the resulting release of mitochondrial apoptogenic factors into the cytosol.

In addition to its interaction with Bax, VDAC1 can also form large enough homo-oligomers that allow the release of cytochrome c and initiate the apoptotic pathway [152]. In this context, VDAC1 has been defined as a pro-apoptotic protein and, for the same reason, it has rapidly become a pharmacological target, especially in cancer research [153]. As HKs modulate VDAC1 activity by controlling the opening/closing of the channel, they also take part in the regulation of apoptosis. By interacting with VDAC1, both HK1 and HK2 reduce its propensity to bind Bax [154]. On the other hand, stimuli that promote HK detachment from mitochondria make the binding site(s) on VDAC1 available for interaction with Bax [155,156].

While no information is available on the role of VDAC3 in apoptosis, the history of VDAC2 is more intricate This isoform was initially referred to as an anti-apoptotic protein. Cheng and colleagues demonstrated its specific ability to sequester Bak into an inactive form and proposed a co-evolution with Bcl-2 proteins, as evidence of their common involvement in the intricated regulation of cell death [157]. The specific domains of VDAC2 were later identified as essential for interaction with Bak [158]. More recently, Chin’s group demonstrated that VDAC2 is essential for mitochondrial recruitment of Bax, a crucial condition for apoptosis activation [159]. However, VDAC2, paradoxically, also ensures the inhibition of Bax itself by mediating its retro-translocation in the cytosol. In this perspective, VDAC2 is a crucial crossroad in regulating the dynamic equilibrium between mitochondrial and cytosolic pools of Bax by responding to the presence of pro- and anti-apoptotic stimuli. Moreover, recent findings suggest that ceramides can trigger apoptosis by binding VDAC2 and blocking Bax retro-translocation [160].

Overall, literature depicts VDACs as key players in cell life and death regulation.

## 8. VDAC as αSyn Binding Partner for Better or for Worse

Recent studies report the ability of αSyn to bind VDAC1 while little is known about the interaction between αSyn and the other isoforms. Data from PLB analysis show that monomeric αSyn reversibly blocks VDAC1 conductance in a voltage-dependent manner already at very low concentrations, and reverses its ion selectivity from anionic to cationic [103]. Furthermore, the C-terminal domain, and not the N-terminal one, would appear to be the specific region of αSyn responsible for this interaction with VDAC1. This could be due to the high content of negatively charged amino acids at the C-terminus of αSyn [103]. Furthermore, it has been shown that VDAC ability to capture αSyn depends on the multiple sub-conformations that αSyn can assume on the membrane surface. The different conformation assumed by αSyn would also appear to respond to the different lipid compositions of the membrane [103,161].

VDAC1 is the main permeation pathway through which calcium is transported from the ER to the mitochondrion and is therefore directly involved in cellular calcium homeostasis [162]. Recently, αSyn has been shown to modulate the VDAC permeability to Ca^2+^. In fact, as a result of αSyn binding, VDAC1 selectivity for calcium increases, which leads to a higher Ca^2+^ flux through the channel [163].

The ability of αSyn to interact with other VDAC isoforms has also been investigated. The affinity of αSyn for VDAC3 is between 10 and 100 times lower than that for VDAC1 [164]. This indicates that, despite the high similarity between the two isoforms, slight differences in cytosolic-exposed domains of VDAC3 could contribute to change the lipid-VDAC interface and, consequently, the ability of the pore to capture αSyn [164]. The different affinity of VDAC3 towards αSyn could be explained by the different content, position, and oxidation pattern of cysteine residues. Cysteines are sensitive to the redox environment and mass spectrometry analyses revealed that VDACs cysteines exist in different oxidation states [125,126]. Utilizing cysteine scanning mutagenesis approach, single and/or multiple mutations of cysteine(s) to alanine have been shown to profoundly change the electrophysiological features of VDAC3 [165]. Furthermore, a cysteine-less VDAC3 shows a different interaction kinetics and requires a higher voltage for αSyn translocation than WT [164]. This result reinforces the concept that cysteines play a prominent role in the regulation of VDAC3 function and allow us to speculate that modification of cysteines’ oxidation state could interfere with the interaction with physiological binding partners [166].

**Figure 2:**
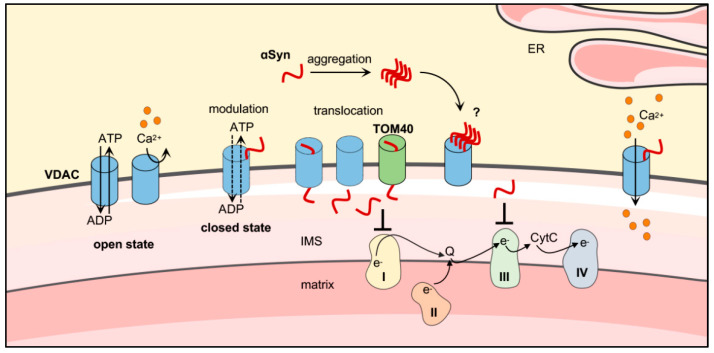
Proposed model of the interaction between αSyn and VDAC on the cytosolic surface of mitochondria. Monomeric αSyn binds VDAC and modulates its electrophysiological activity promoting the transition from the open to the closed state. No information is available so far about αSyn oligomers or fibrils and VDAC. The OMM β-barrel proteins, VDAC and TOM40, promote the αSyn translocation from cytosol to IMS of mitochondria, where it is believed responsible of the impairment of electron transport system complex I/III activity. Moreover, αSyn is involved in the modulation of calcium influx into mitochondria from endoplasmic reticulum (ER). Original figure created with Smart Servier Medical Art tools (https://smart.servier.com, 2021).

Little is known about the relationship between pathological species of αSyn and VDAC. Using a proteomic approach combined with in vitro pull-down experiments of mitochondrial extracts and mass spectrometry analysis, McFarland and colleagues highlighted the specific differences between the phosphorylated at Ser 129 or Tyr 125 and the unphosphorylated C-terminus interactome of αSyn [167]. Specifically, they reported that unphosphorylated C-terminus preferentially binds mitochondrial proteins including TOM22, TOM40, all three VDAC isoforms and Sam50. However, while phosphorylation of S129 drastically reduces the association of αSyn with TOM40 and Sam50, no difference in interaction was observed with VDACs [167]. Recently, the interactome of docosahexaenoic acid-stabilized αSyn oligomers from rat primary hippocampal neurons has been analyzed through a proteomic approach in which photo-crosslinking of labelled αSyn bound to bait proteins preceded the cell lysis. As a result, 32 binding partners of αSyn oligomers were reported and, not surprisingly, most of them were membrane-bound or membrane-associated proteins. In this report, VDAC was listed as a unique family of mitochondrial protein [168]. In the same work, a computational prediction of the binding sites of αSyn oligomers was performed. In the case of VDAC, the loops facing the cytosol represent the ‘hot spots’ of interaction [168]. A schematic representation of the interaction between αSyn and VDAC is shown in Figure 2**.**

Direct αSyn-VDAC interaction has been reported in the SNpc of rats over-expressing αSyn [169]. In this model, the synucleinopathy-like pathology led to a loss of DA neurons and mitochondrial dysfunction. In particular, swollen mitochondria and loss of mitochondrial membrane integrity were observed, suggesting that increased permeabilization had occurred [169]. These data were related to the opening of the mitochondrial permeability transition pore (PTP), which leads to bioenergetics failure and/or mitochondria-mediated apoptosis. PTP is a multiprotein complex whose exact molecular composition is still debated. According to several studies, the putative scaffold of PTP would be constituted by the dynamic interaction between VDAC and the adenine nucleotide translocator (ANT) in the outer and inner mitochondrial membranes respectively. Its opening is modulated by cyclophilin D (CypD), Ca^2+^, pro-oxidant agents and several VDAC- and/or ANT-binding partners (e.g., Bax, Bak, Bid, HK) [170]. In this perspective, Martin and colleagues demonstrated an association between mitochondrial abnormalities in PD and impairment of PTP. Specifically, transgenic mice overexpressing the αSyn A53T mutant showed a correlation between apoptotic cell death induction in the SNpc and mitochondrial swelling in neurons positive for αSyn. The latter finding was indicative of increased mitochondrial permeabilization. Furthermore, αSyn A53T was shown to bind VDAC and CypD *in vivo*. Genetic deletion of CypD, the main regulator of PTP, delayed disease onset and extended mice survival, providing strong evidence for the involvement of PTP-related mechanisms in PD progression [171]. More recent data also show that the oxidative insult due to exogenously applied αSyn oligomers increases sensitivity to PTP opening, leading to mitochondrial swelling and ultimately cell death [100].

Overall, these data indicate that αSyn has an “unsuspected” physiological role in controlling mitochondrial Ca^2+^ fluxes and mitochondrial bioenergetics by regulating the function of VDAC1/3 and the permeability of VDAC1 to oppositely charged ions. Conversely, changes in the cellular environment such as aggregation and accumulation of αSyn, the lipid composition of the OMM and the potential across the OMM may render αSyn–VDAC interaction detrimental, thus contributing to trigger mitochondrial dysfunction and abnormal cell death in a pathological context.

## 9. VDAC Levels in PD

Changes in cellular VDAC levels have been reported in numerous stress and pathological conditions, including cancer and neurodegenerative disorders [122,172,173]. To date, most studies have focused on Alzheimer’s disease and partly on ALS, and little is known about variations VDAC expression in PD. For instance, Chu and colleagues have shown that VDAC1 expression was significantly reduced in nigral neurons of PD patients and in rat models overexpressing αSyn A30P mutant [174]. They also suggested an association between reduced VDAC1 levels and αSyn aggregation in both experimental and sporadic PD. In agreement with these findings, another study reported a robust decrease in VDAC1 and VDAC2 levels in SNpc samples from PD patients and SH-SY5Y neuroblastoma cell line treated with dopamine [175]. As mentioned above, dysregulation of dopamine oxidative metabolism causes the generation of pro-oxidant species that contribute to impaired mitochondrial function in PD [176]. The dopamine-induced decrease in VDAC levels was specifically attributed to increased protein degradation by mitochondrial proteases and the alteration of calcium influx, mitochondrial potential and network integrity [175]. Conversely, rotenone or MPP^+^ treatment increased VDAC1 and VDAC2 protein levels by approximately 50% in SH-SY5Y cells [175,177]. Increased expression of VDAC1 has also been reported in the striatum and cortex of Parkin KO mice [178] and in extracts from 6-hydroxydopamine lesion [81], a pro-oxidant derivate of dopamine commonly used to model ROS-induced cell death. It is conceivable that the discrepancies in the information obtained on VDAC levels are probably due to PD models used, the intrinsic heterogeneity of the PD phenotype as well as the stage of the disease.

Interestingly, the mRNA for αSyn and VDAC1 are both common targets of microRNA-7 (miR-7) [179], indicating a common control of their respective expression levels. MicroRNAs (miRNAs) are a class of small non-coding RNAs (ncRNAs) which suppresses the translation and/or promotes the degradation of target mRNAs by binding to their 3′-untranslated region (3′-UTR). Altered expression of specific miRNAs has been linked to the pathogenesis of PD [180]. In particular, in the SNpc of PD patients and MPTP-induced parkinsonian mice, significant miR-7 depletion has been associated with over-expression and accumulation of αSyn, dopaminergic neurodegeneration and reduced dopamine secretion in the striatum [181]. It has also been suggested that in the MPP^+^ model, the neurotoxin-induced increase in VDAC1 levels in SH-SY5Y cells is the result of miR-7 down-regulation [182]. Moreover, silencing VDAC1 expression by miR-7 preserves mitochondrial function after MPP^+^ treatment by preventing depolarization of mitochondria and attenuating the initiation of apoptotic events [182].

Together, these studies reveal that miR-7 attenuates the neurotoxic effects of MPP^+^ and that the increase of αSyn and VDAC1 levels caused by miR-7 down-regulation could be related to mitochondrial dysfunction, suggesting that the increased VDAC1 level could be used as an early marker of cell death in PD.

## 10. VDAC as a Promising Therapeutic Target in Disease Treatment

Given the central role of VDAC in the control of metabolic fluxes across the OMM and the intrinsic apoptosis pathway, the protein has been proposed as a potential pharmaceutical target in the treatment of several diseases, including cancer and neurodegenerative disorders. For example, VDAC1 has been successfully used as a template for the design of synthetic peptides based on VDAC and/or binding partners aimed at interfering with the aggregation of misfolded proteins on the cytosolic surface of mitochondria in ALS [145,146]. A similar strategy is conceivable also in the case of αSyn. In recent years, the cholesterol-like compound olesoxime has emerged as a drug candidate for the treatment of neurodegenerative disorders [183]. The neuroprotective effect of olesoxime is mainly attributed to its ability to bind mitochondria through interaction with VDAC or the translocator protein (TSPO) [104,184]. Therefore, its beneficial properties are probably associated with the functions of these two proteins. This compound leads to inhibition of PTP, reduction of oxidative stress, and stabilization of cholesterol homeostasis [104,183]. Interestingly, olesoxime ability to counteract mitochondrial toxicity has also been reported in SHSY-5Y cells overexpressing αSyn [185], as demonstrated by the reduction of cytochrome c release into the cytosol and activation of caspases. Moreover, olesoxime also reduces αSyn-induced dissipation of the mitochondrial membrane [104].

Rostovtseva’s group has shown that the compound acts directly on VDAC1. In particular, olesoxime modified the typical voltage dependency behavior when reconstituted at the PLB, and prevented the translocation of αSyn to the IMS [104].

Curcumin has also been indicated as a potential pharmacological tool in this context. Indeed, oligomeric or fibrillar forms of αSyn appear to play a crucial role in the release of HK1 from the mitochondria, triggering a pro-apoptotic signal. As the N-terminus of HK modulates the VDAC activity, closure and gating of the channel together with the protective effect exerted by curcumin may closely be linked to the maintenance of VDAC-HK complexes [186].

## 11. Conclusions

Treatment of mitochondrial dysfunction in neurodegenerative diseases is one of the most discussed and interesting challenge in recent literature, as the health of these organelles is essential for the maintenance of several cellular functions, especially in neurons, where high levels of ATP are required. Being the main mitochondrial gate for substrates and a hub for many proteins in both physiological and pathological conditions, VDAC represents a promising target for the design of molecules aimed at interfering with αSyn interaction at the mitochondrial surface and/or its translocation through OMM. Although much remains to be elucidated, modulating VDAC activity may be a winning strategy to significantly ameliorate the mitochondrial pathology and to counteract the toxic effect exerted by αSyn and other misfolded proteins, at least at the organelle level.
